# Effect of High-Temperature Storage on Electrical Characteristics of Hydrogen-Treated AlGaN/GaN High-Electron-Mobility Transistors

**DOI:** 10.3390/mi15050611

**Published:** 2024-04-30

**Authors:** Bin Zhou, Chang Liu, Chenrun Guo, Xianghong Hu, Xiaodong Jian, Hongyue Wang, Xiaofeng Yang

**Affiliations:** 1 The Fifth Electronics Research Institute of the Ministry of Industry and Information Technology, Guangzhou 511370, China; 2 National and Local Joint Engineering Research Center of Reliability Analysis and Testing Technology of Electronic Information Products, Guangzhou 511370, China

**Keywords:** AlGaN/GaN HEMT, hydrogen, high-temperature storage

## Abstract

In this paper, high-temperature storage of hydrogen-treated AlGaN/GaN HEMTs is conducted for the first time to study the effect of high temperature on the electrical characteristics of the devices after hydrogen treatment, and it is found that high-temperature storage can effectively reduce the impact of hydrogen on the devices. After hydrogen treatment, the output current and the maximum transconductance of the device increase, and the threshold voltage drifts negatively. However, after high-temperature treatment at 200 °C for 24 h, the output current, threshold voltage, and the maximum transconductance of the device all approach their initial values before hydrogen treatment. By using low-frequency noise analysis technology, the trap density of the hydrogen-treated AlGaN/GaN HEMT is determined to be 8.9 × 10^23^ cm^−3^·eV^−1^, while it changes to 4.46 × 10^22^ cm^−3^·eV^−1^ after high-temperature storage. We believe that the change in the electrical characteristics of the device in hydrogen is due to the passivation of hydrogen on the inherent trap of the device, and the variation in the electrical properties of the device in the process of high-temperature storage involves the influence of two effects, namely the dehydrogenation effect and the improvement of the metal–semiconductor interface caused by high temperatures.

## 1. Introduction

Due to the advantages of the wide bandgap, high breakdown voltage, high concentration of two-dimensional electron gas (2DEG), excellent thermal conductivity, and high electron mobility, GaN-based high-electron-mobility transistors (HEMTs) have great application prospects in high-temperature, high-frequency, and aerospace fields [1,2,3]. For hermetic-sealing devices in aerospace and other applications, hydrogen is an environmental factor that can affect reliability. Since the 1980s, hydrogen has been found to have an impact on the electrical characteristics of compound semiconductor devices. In 1989, the degradation of GaAs FETs and MMIC due to the presence of hydrogen was reported [4]. Studies have shown that hydrogen as low as 0.5% can also cause significant degradation of the device at higher temperatures (125 °C) and in a relatively short time (168 h). P. C. Chao et al. [5] reported that the GaAs PHEMTs with Ti/Pt/Au gate decreased in transconductance and output current at 270 °C and a 4% hydrogen concentration, while the pinch-off voltage increased and the source resistance remained unchanged. In 1994, a lifetime model of the hydrogen effect was obtained, which showed that the degradation was related to the hydrogen partial pressure and temperature [6]. In the meantime, researchers began to pay attention to the interaction between hydrogen and GaN materials, focusing on the form of hydrogen in GaN and its effects on impurities [7,8]. Then, GaN-based hydrogen sensors were manufactured and demonstrated favorable hydrogen sensitivity [9,10,11]. In recent years, the impact of hydrogen on the reliability of GaN devices has become a new concern [12,13,14,15]. The hydrogen in GaN devices mainly comes from the process of material preparation and package assembly. During the preparation process of AlGaN/GaN HEMTs, hydrogen is introduced into the material through metal organic chemical vapor deposition (MOCVD) or hydride vapor phase epitaxy (HVPE) methods [16]. The MOCVD method needs hydrogen as the carrier gas to promote the growth of AlGaN and GaN, and the HVPE method requires hydrogen as a reducing agent to reduce the metal ions in the preparation reaction to metal atoms. Both the MOCVD and HVPE methods introduce hydrogen into AlGaN/GaN materials. Moreover, hydrogen is also introduced in packaging processes such as electroplating and is absorbed by the shell material. For AlGaN/GaN HEMTs using hermetic-sealing technology, hydrogen can be released from the AlGaN/GaN HEMTs materials and packaging material, accumulating in the sealed cavity and leading to reliability issues and changes in the electrical characteristics [17]. Previous studies have shown that exposing AlGaN/GaN HEMTs to a hydrogen environment can increase the gate leakage current by several orders of magnitude and significantly reduce electron mobility in the channel [18]. It has also been found that hydrogen can passivate traps inside devices, resulting in threshold voltage shifts and increased saturation output currents [12]. Hydrogen-treated devices may also be more susceptible to other stresses; for example, it has been found that the degradation effect of protons on hydrogen-treated devices is more severe [19]. In order to understand whether the hydrogen-treated device can be recovered in a high-temperature environment, the high-temperature storage of hydrogen-treated AlGaN/GaN HEMTs is conducted, and the DC, C–V and pulse I–V characteristics before and after storage are compared. Based on the measurement results of the low-frequency noise, the mechanism of the high-temperature storage of hydrogen-treated AlGaN/GaN HEMTs is obtained.

## 2. Materials and Methods

**Figure 1:**
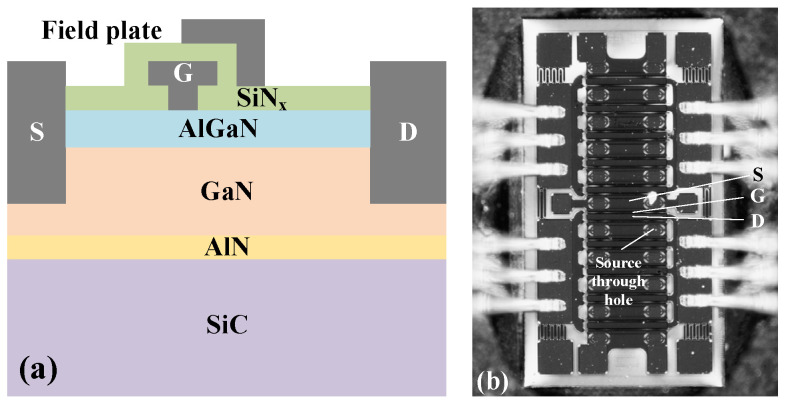
Device structure: (**a**) the schematic diagram of the cross-section and (**b**) surface morphology of the AlGaN/GaN HEMTs.

The schematic diagram and the surface morphology of the device are shown in Figure 1. The SiN_x_ protective layer thickness is approximately 0.15 μm. The thickness of the metal gate deposited on the GaN material layer is approximately 0.15 μm. The thickness of the AlGaN barrier layer is 25 nm, and the thickness of the GaN buffer layer is 2 μm. The thickness of the Si substrate at the bottom is 350 μm, the gate width (*W*_g_) of the AlGaN/GaN HEMTs is 1.25 mm, and the length (*L*_g_) is 0.5 μm.

**Figure 2:**
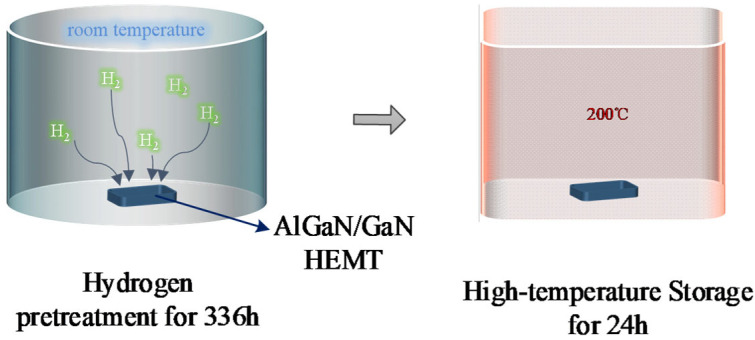
Schematic diagram of the high-temperature storage experiment for hydrogen-treated AlGaN/GaN HEMTs.

The specific experimental process is shown in Figure 2. Firstly, the AlGaN/GaN HEMTs were placed in a sealed chamber filled with hydrogen–nitrogen mixture gas at standard atmospheric pressure and room temperature. Due to the safety requirements of the laboratory, the hydrogen–nitrogen mixture gas was composed of 96% N_2_ and 4% H_2_. The devices were maintained in this environment for 336 h to allow hydrogen to fully contact the devices. The hydrogen-treated AlGaN/GaN HEMTs were then transferred to an environmental test chamber and kept at 200 °C for 24 h. Before and after the hydrogen treatment and high-temperature storage, the DC, C–V and pulsed I–V characteristics of the devices were measured using a semiconductor parameter analyzer (Agilent B1500A, Keysight, Santa Rosa, CA, USA). In addition, the noise power spectral density of the devices was extracted using a low-frequency noise-testing system consisting of Keysight E4727A and Agilent B1500A (Keysight, Santa Rosa, CA, USA).

## 3. Results

### 3.1. DC Characteristics

**Figure 3:**
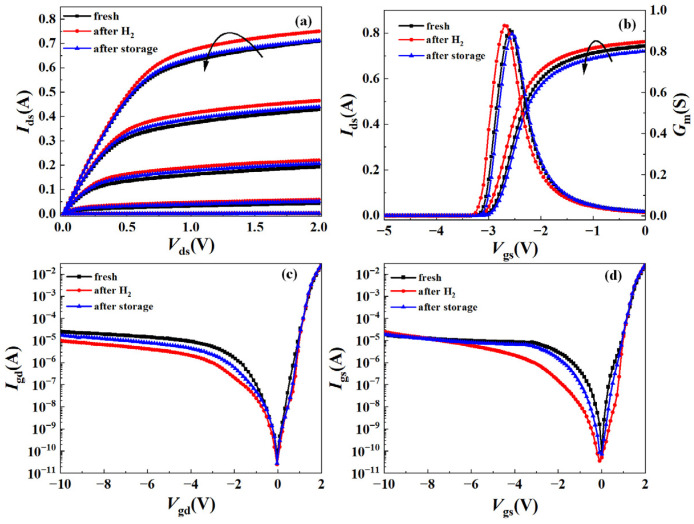
DC characteristics of the fresh device, the hydrogen-treated device and the device after high-temperature storage: (**a**) transfer characteristics, (**b**) output characteristics, (**c**) gate-to-drain leakage current characteristics, and (**d**) gate-to-source leakage current characteristics.

To determine the effect of high-temperature storage on the DC characteristics of the hydrogen-treated AlGaN/GaN HEMTs, we compared the transfer and output characteristics of the fresh device, the hydrogen-treated device and the device after high-temperature storage, as shown in Figure 3a,b. Figure 3a shows the variation trend of the output characteristics of the AlGaN/GaN HEMT with *V*_gs_ ranging from −3.2 V to −2.4 V (0.2 V step). After the hydrogen treatment, the output saturation current of the device significantly increased, while after high-temperature storage, the output saturation current decreased and returned to the initial value. When measuring the transfer characteristics, the *V*_ds_ is specified as 0.5 V. As shown in Figure 3b, after the hydrogen treatment, both the transfer curve and transconductance curve of the device drifted negatively. When the device was in the on-state, the *I*_ds_ of the hydrogen-treated device was greater than the original value at the same *V*_ds_ and *V*_gs_. However, after high-temperature storage at 200 °C for 24 h, the transfer curve of the device drifted positively and approached its initial value, which was consistent with the output curve. We also extracted the maximum transconductance (*G*_mmax_) from the transconductance curve. It was found that the *G*_mmax_ of the device increased from the initial 0.9S to 0.93S after the hydrogen treatment. After high-temperature storage, the *G*_mmax_ of the device returned to 0.89S, which was closer to the initial value.

In order to analyze the effect of the hydrogen treatment and high-temperature storage on the Schottky junction and gate electrode of the AlGaN/GaN HEMTs, the gate-to-drain leakage current (*I*_gd_) and gate-to-source leakage current (*I*_gs_) curves of the AlGaN/GaN HEMTs were obtained, as shown in Figure 3c,d. It can be seen that the influence of hydrogen on the *I*_gs_ mainly occurred near the threshold voltage. After the hydrogen treatment, both the *I*_gs_ and *I*_gd_ of the devices decreased by an order of magnitude, but after high-temperature storage, the recovery phenomenon of the *I*_gs_ and *I*_gd_ appeared to varying degrees in the device.

**Figure 4:**
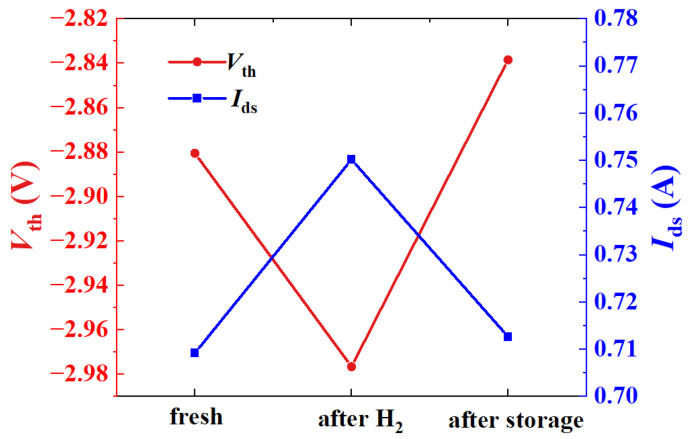
*V*_th_ and *I*_ds_ of AlGaN/GaN HEMTs after hydrogen treatment and high-temperature storage.

Figure 4 shows the changes in the threshold voltage (*V*_th_) and the *I*_ds_ (under test conditions of *V*_gs_ = −2.4 V and *V*_ds_ = 2 V) in the different experimental phases visually. From Figure 4, the *V*_th_ of the AlGaN/GaN HEMT changed from the initial −2.88 V to −3.00 V after the hydrogen treatment. Then, it returned to its initial value after high-temperature storage. The *I*_ds_ of the AlGaN/GaN HEMT showed same trend. It increased by 41 mA from the initial 709 mA to 750 mA after the hydrogen treatment. Then, the *I*_ds_ decreased to 712 mA after high-temperature storage, which was close to the initial value. The change of parameters after the hydrogen treatment may be attributed to the passivation of the inherent defects within the device by hydrogen [20,21]. After high-temperature storage, hydrogen gradually resolved from the device, causing the *V*_th_ and *I*_ds_ to return to the vicinity of the initial value.

### 3.2. C–V and Pulsed I–V Characteristics

**Figure 5:**
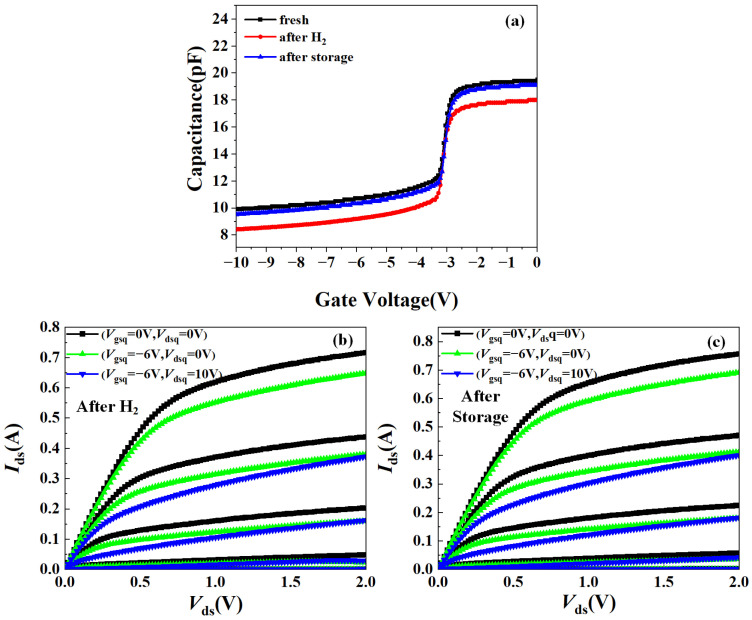
C–V and pulse I–V characteristics of AlGaN/GaN HEMTs after hydrogen treatment and high-temperature storage: (**a**) C–V characteristics; (**b**) pulse I–V characteristics of the hydrogen-treated device; and (**c**) pulse I–V characteristics of the device after high-temperature storage.

Figure 5a compares the C–V characteristics of the AlGaN/GaN HEMTs before and after the hydrogen treatment, as well as after 200 °C storage, at a frequency of 1 MHz. It can be seen that the barrier capacitance of the AlGaN/GaN HEMT reduced after the hydrogen treatment but increased to its initial value after high-temperature storage.

**Figure 6:**
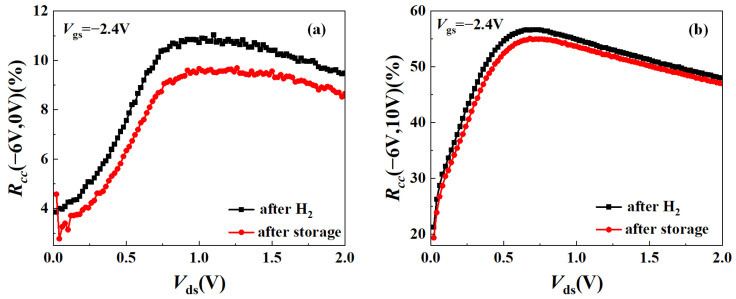
Comparison of current collapse rate after hydrogen treatment and high-temperature storage: (**a**) the off-state is (*V*_gsq_, *V*_dsq_) = (−6 V, 0 V); and (**b**) the off-state is (*V*_gsq_, *V*_dsq_) = (−6 V, 10 V).

To characterize the current collapse effect of the hydrogen-treated AlGaN/GaN HEMTs before and after high-temperature storage, pulsed I–V measurements were performed on the devices. The current collapse effect is related to electron transport and capture during each static bias period, and it can reflect the trap density and interface state density in the barrier layer [22]. We selected three different static bias points, (*V*_gsq_, *V*_dsq_) = (0 V, 0 V), (−6 V, 0 V), and (−6 V, 10 V). The pulse width was set to 5 μs and the pulse interval was set to 10 ms. When (*V*_gsq_, *V*_dsq_) = (0 V, 0 V), no stress was applied to the gate and drain of the device, so there was no obvious current collapse phenomenon. However, when the device was in the off-state at (*V*_gsq_, *V*_dsq_) = (−6 V, 0 V) and (−6 V, 10 V), applying a voltage would cause the charge and discharge phenomena of the traps in the material. Figure 5b,c show the pulsed I–V output characteristics of the hydrogen-treated AlGaN/GaN HEMTs before and after high-temperature storage. In order to observe the current collapse of the device more accurately, a formula was used to quantify the current collapse rate *R*_cc_ of the device [23].

(1) $$R_{\mathrm{cc}}=\frac{I_{\mathrm{ds}}(V_{\mathrm{gsq}}=0\;V,V_{\mathrm{dsq}}=0\;V)-I_{\mathrm{ds}}(V_{\mathrm{gsqi}},V_{\mathrm{dsqi}})}{I_{\mathrm{ds}}(V_{\mathrm{gsq}}=0\;V,V_{\mathrm{dsq}}=0\;V)},$$

where *V*_gsqi_ and *V*_dsqi_ are the static voltage biases that make the device in the off-state. According to (1), the *R*_cc_ of the hydrogen-treated AlGaN/GaN HEMT before and after high-temperature storage was extracted. Using the *I*_ds_ at *V*_gs_ = −2.4 V as the basis for comparison, the current collapse rates under the two off-state bias were obtained and are shown in Figure 6a,b.

Under the static bias conditions of (*V*_gsq_, *V*_dsq_) = (−6 V, 0 V) and (−6 V, 10 V), the *R*_cc_ of the hydrogen-treated AlGaN/GaN HEMTs at *V*_ds_ = 2 V, *V*_gs_ = −2.4 V decreased from 10.4% and 50.21% to 8.7% and 47.09% after high-temperature storage, respectively, indicating that the effect of the traps capturing channel electrons was weakened. This phenomenon may be due to the reduction in the interface state density in the barrier layer after high-temperature storage.

### 3.3. Low-Frequency Noise Characteristics

**Figure 7:**
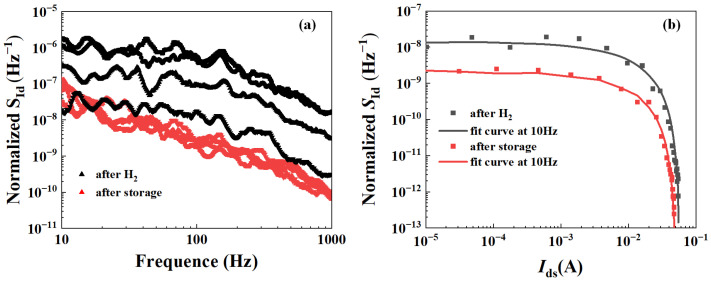
The characteristics of low-frequency noise for AlGaN/GaN HEMTs: (**a**) *S*_Id_/I^2^ versus frequency for the hydrogen treatment devices before and after storage; and (**b**) *S*_Id_/*I*^2^ versus *I*_DS_ at 10 Hz for the hydrogen-treated devices before and after storage.

To verify that high-temperature storage can reduce the density of the traps in hydrogen-treated AlGaN/GaN HEMTs, the trap density of the hydrogen-treaded devices before and after storage was analyzed by using low-frequency noise technology. Under a drain bias of 100 mV, a low-frequency noise analysis system was used to measure the noise power spectral density (*S*_Id_) of the hydrogen-treated devices before and after high-temperature storage in the frequency range of 10 Hz to 1000 Hz, as shown in Figure 7. From Figure 7a, it can be seen that the *S*_Id_ has a good correlation with the 1/*f* rule in the tested frequency range.

In order to further analyze the low-frequency noise characteristics, we constructed the curve of the *S*_Id_/*I*_ds_^2^ and (*G*_m_/*I*_ds_)^2^ with the *I*_ds_, as shown in Figure 7b. It was found that they had a good consistency, indicating that the low-frequency noise of the device conformed to the carrier number fluctuation model [24,25]. Therefore, the flat-band voltage noise power spectral density *S*_vfb_ can be calculated according to (2) [26,27].

(2) $$\frac{S_{\mathrm{Id}}}{I_{\mathrm{ds}}^{2}}={\left(\frac{G_{m}}{I_{ds}}\right)}^{2}S_{vfb},$$

The *S*_vfb_ of the hydrogen-treated AlGaN/GaN HEMTs before and after storage were 1.88 × 10^−11^ Hz^−1^ and 9.79 × 10^−13^ Hz^−1^, respectively. The trap density (*N*_ot_) in the AlGaN/GaN HEMTs can be determined by the following formula [28,29].

(3) $$N_{ot}=\frac{WLfC_{b}^{2}}{q^{2}k_{B}T\lambda }S_{vfb},$$

where *q* is the electronic charge, *k*_B_ is the Boltzmann constant, *T* is the absolute temperature, *λ* = 0.5 is the tunneling parameter, corrected as AlGaN/GaN conduction band alignment here, *W* and *L* are the gate width and length, *f* is the frequency, and *C*_b_ is the AlGaN barrier capacitance. According to (3), the extracted *N*_ot_ were 8.9 × 10^23^ eV^−1^·cm^−3^ and 4.46 × 10^22^ eV^−1^·cm^−3^ before and after the storage. It can be seen that high-temperature storage treatment can effectively reduce the trap density of hydrogen-treated AlGaN/GaN HEMTs.

## 4. Discussion

**Figure 8:**
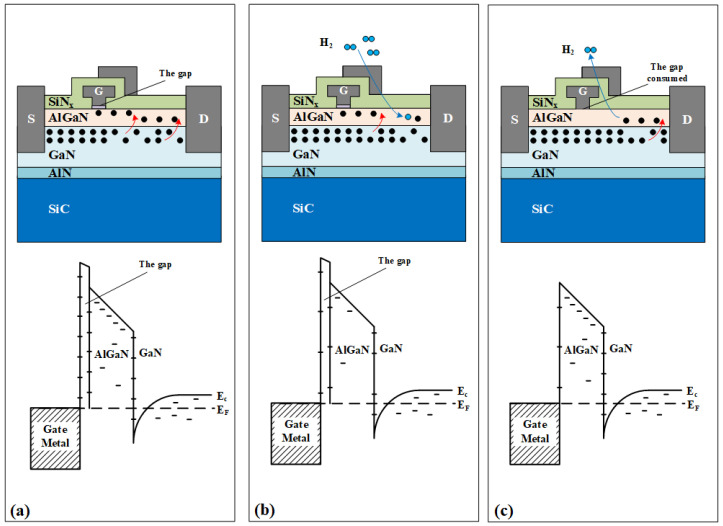
Evolution of the AlGaN/GaN HEMT band scheme: (**a**) fresh devices, (**b**) the devices after hydrogen treatment, and (**c**) the devices after high-temperature storage.

The mechanism is illustrated in Figure 8. During hydrogen treatment, it is observed that the drain current of the device increases, the threshold voltage negatively drifts and the gate leakage current decreases, which is mainly due to the passivation effect of hydrogen on the inherent trap of GaN devices. As shown in Figure 8a, there are lots of defects in the GaN buffer layer, AlGaN barrier layer, heterostructure interface, the gap layer and AlGaN surface of the AlGaN/GaN HEMTs because of the heteroepitaxy process for the current state of the art device, including the Ga vacancies (*V*_Ga_), nitrogen antisites (*N*_Ga_), and N vacancies (*V*_N_) [30,31]. These defects assist electron tunneling and trap charge carriers, resulting in current collapse and large noise power spectral density. Previous studies have shown that hydrogen will be catalyzed by the gate metal Pt, from hydrogen molecules to hydrogen atoms, so as to move through the SiN_x_ passivation layer and enter the device [4,5,12,32,33]. These hydrogen atoms interact with these inherent defects, forming hydrogenation defects such as [*V*_Ga_*H*_3_], [*N*_Ga_*H*_2_], and [*V*_N_*H*_2_] [16,20]. This effect reduces the total number of defects inside the device so that these defects no longer capture electrons, thus increasing the channel carrier concentration and further increasing the drain current.

After high-temperature storage, the situation is different. The degradation mode of the device shows that the drain current decreases and the threshold voltage positively drifts compared with the hydrogen-treated device. We believe that this is the comprehensive result of dehydrogenation effect [34] and the improvement of the metal–semiconductor interface caused by storage [35]. First, previous studies have shown that hydrogen-containing compounds are unstable and prone to dehydrogenation at high temperatures [5]. After dehydrogenation of the hydrogenation defects such as [*V*_Ga_*H*_3_], [*N*_Ga_*H*_2_], and [*V*_N_*H*_2_] described above, the trap density in the device will increase, but this is clearly the opposite of the measurement results for the current collapse and low-frequency noise. Therefore, we believe that high-temperature storage also improves the quality of the metal–semiconductor contact in the device. After the device is manufactured, the contact between the metal and the semiconductor is not tight, as there is a gap of several nanometers [36,37], but after high-temperature storage, this gap is consumed, forming a tighter metal–semiconductor contact interface [35,38]. This results in a decrease in the trap density at the Schottky contact interface, counteracting the increase in the trap density due to dehydrogenation. As we know, the current collapse effect is related to the trap on the device surface, and the low-frequency noise characterizes the average trap density near the Fermi level. Therefore, with the improvement of the metal–semiconductor interface, the current collapse effect is weakened and the trap density measured by low-frequency noise is also reduced.

## 5. Conclusions

In summary, hydrogen can affect the electrical characteristics of AlGaN/GaN HEMTs, resulting in negative threshold voltage drift and increased drain current. By storing the hydrogen-treated device at a high temperature of 200 °C for 24 h, the threshold voltage and drain output current of the device regress to the typical initial values. The change in the electrical characteristics of the device in hydrogen is due to the passivation of hydrogen on the inherent trap of the device, and the variation in the electrical properties of the device in the process of high-temperature storage involves the influence of two effects, namely the dehydrogenation effect and the improvement of the metal–semiconductor interface caused by storage at high temperatures.

## Data Availability

The original contributions presented in the study are included in the article, further inquiries can be directed to the corresponding authors.
